# Atypical Presentation of Giant Mandibular Aneurysmal Bone Cyst with Cemento-Ossifying Fibroma Mimicking Sarcoma

**DOI:** 10.1155/2019/1493702

**Published:** 2019-08-27

**Authors:** Haneen Al-Maghrabi, Serge Verne, Bassam Al-Maghrabi, Osamah Almutawa, Jaudah Al-Maghrabi

**Affiliations:** ^1^Department of Pathology, King Faisal Specialist Hospital and Research Center, Jeddah, Saudi Arabia; ^2^Oral and Maxillofacial Surgery, King Abdulaziz Hospital/Ministry of National Guard Health Authority, King Faisal Specialist Hospital and Research Center, Jeddah, Saudi Arabia; ^3^Faculty of Dentistry, King Abdulaziz University, Jeddah, Saudi Arabia; ^4^College of Medicine, King Khalid University, Abha, Saudi Arabia; ^5^Department of Pathology, King Abdulaziz University, Jeddah, Saudi Arabia

## Abstract

Aneurysmal bone cysts (ABCs) are an uncommon osteolytic intraosseous bone lesions. ABCs commonly involve metaphysis of long bones and are rarely diagnosed in craniofacial bones. The World Health Organization (WHO) designates ABCs as benign, but locally destructive, rapidly growing masses. ABC can be clinically misdiagnosed as a malignant tumor. In this article, we present a case of a 12-year-old female patient who presented to a maxillofascial outpatient clinic complaining of huge jaw swelling over the past 3 months, which was clinically suggestive of sarcoma. Few reported cases in the English literature describe ABC presented with huge rapidly growing mass causing destructive bony lesion that was clinically mimic sarcoma, which initiates this case report. We also discuss the most important pathologic differential diagnosis of tumors with malignant behavior and review the literature.

## 1. Introduction

An aneurysmal bone cyst (ABC) is a benign osteolytic, rapidly growing, expansive, hemorrhagic bone lesion. ABCs favor long bones, such as the femur, tibia (up to 50%), and spine (12–30%) [1]. Craniofacial bones are rarely the site of ABCs (accounting for 2–12% of all ABCs) [2]. The mandible is more commonly involved than the maxilla, with an estimated ratio of 2 : 1 to 11 : 9 [3]. The body of the mandible and ramus are the most commonly involved regions. Few published papers have reported the involvement of the coronoid process and mandibular condyle. One study estimated that 90% of all ABCs affect the posterior mandible, as follows: body of the mandible (40%), ramus (30%), angle (19%), symphysis (9%), and condyles (2%) [4]. Few cases reported in the literature have stated that a jaw ABC crosses the midline [5]. The first two decades of life are the most common age of presentation. The median age of diagnosis is 13 years old. Some studies favor a slight female preponderance [1, 4], while others show no sex predilection [6]. Clinical signs and symptoms are not specific for ABC, which might cause difficulty in the accurate diagnosis. Only a few have presented with a rapidly growing mass and a destructive bony lesion that can mimic sarcoma or high-grade malignancy. This rapid growth can be explained by the erosion of cortical plates. Thus, the clinical diagnosis should be based on patient presentation, imaging studies, and histopathological examinations. However, histopathology diagnosis can be difficult to assess on needle tissue biopsy. In this paper, we will present a rare case of a huge jaw mass in a 12-year-old girl, destroying half of her mandible, clinically mimicking high-grade sarcoma. Pathogenesis, clinical manifestation, and differential diagnosis will also be discussed.

## 2. Case Presentation

**Figure 1:**
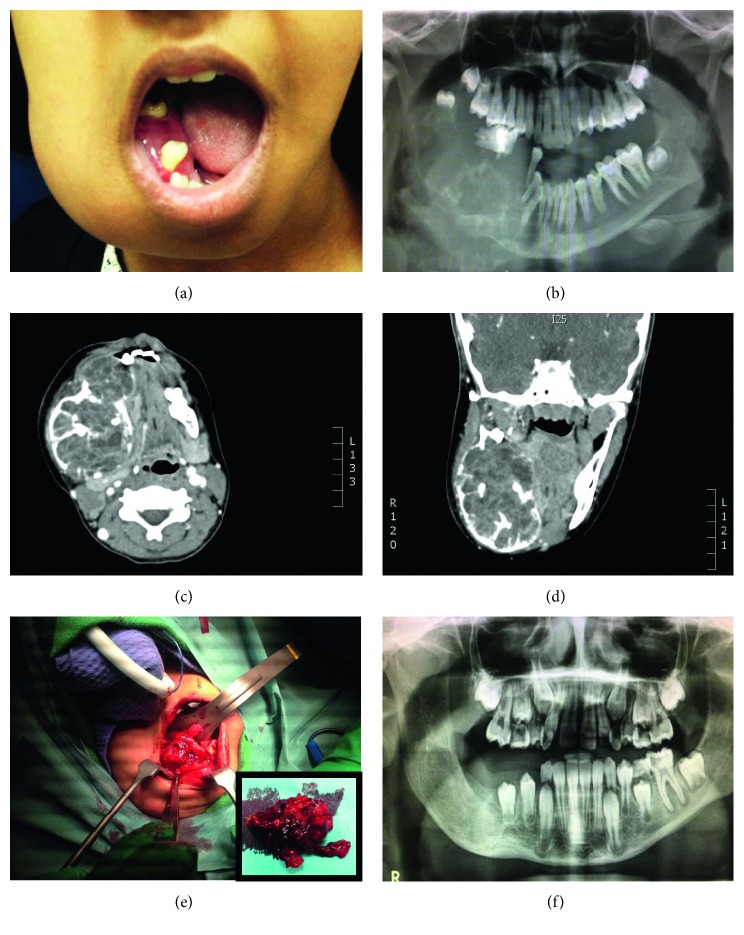
(a) A 12-year-old girl with right mandibular and neck mass displacing teeth all the way. (b) Panorex view that shows massive destruction of half of her mandible with extension into condyle, neck, and medial of right of mandible. (c) Axial and (d) coronal CT scan of neck and soft tissue that shows multiple septations along with wall enhancement within the lesion. (e) Large soft tissue tumor was visible intraoperatively. Gross examination of large hemorrhagic soft tissue mass measures 6 × 6 × 3 cm (inset). (f) Postoperative panorex view of the patient which shows complete healing of her bony defect with significant resolution in her unique facial deformity that resembles sarcoma.

**Figure 2:**
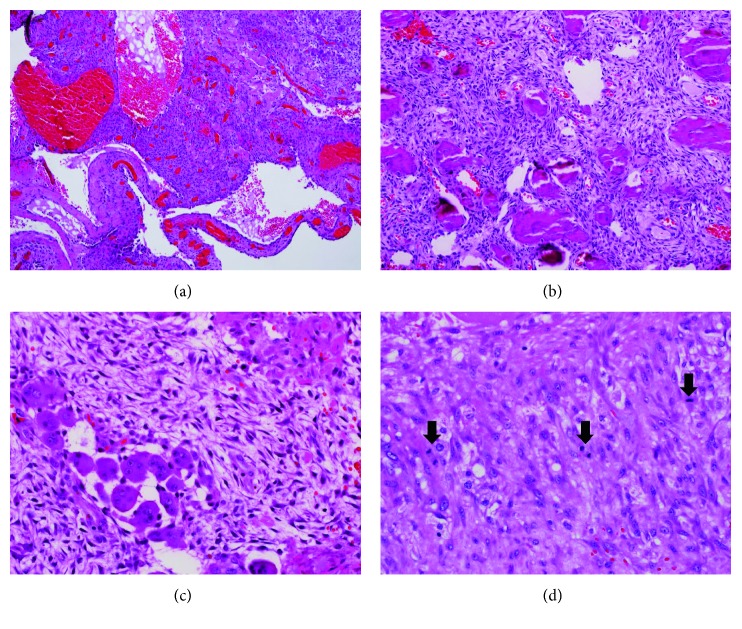
Histopathology examination by hematoxylin and eosin stain (H&E). (a) Tumor is composed of spaces separated by septa (blood-filled cavities) (H&E; 4x). (b) Spindle cell proliferation with classification and osteoid formation (H&E; 10x). (c) Cellular components with osteoclast-like giant cells (H&E; 40x). (d) Numerous mitotic figures are seen (arrow head) (H&E; 40x).

This is a 12-year-old girl who was referred from an outside hospital as a case of right mandibular and neck mass, which was rapidly progressive for the last 3 months (Figure 1(a)). The patient had a computerized tomography (CT) scan done outside, which revealed a destructive mandibular mass. The outside incisional biopsy was inadequate and showed changes suggestive of cemento-ossifying fibroma. The patient was subsequently admitted to the King Faisal Specialist Hospital and Research Center. Her physical examination shows stable vital signs with mild trachea displaced to the left by this large destructive mass. Repeated CT scan of the mandible and neck soft tissue showed a very large expansile lytic mass (7.3 cm craniocaudal × 5.1 cm transverse × 8.2 cm AP) involving the right side of the mandible, causing massive destruction of half of her mandible with extension into her condyle, neck, and medial side of the right mandible (Figure 1(b)). Areas of septations and bony erosions were seen peripherally. The enhanced study revealed multiple septations that contain wall enhancement within the lesion (Figures 1(c) and 1(d)). The clinical diagnosis was high-grade malignancy, such as Ewing sarcoma. No significant major lymph node enlargement was detected. Visualized brain parenchyma showed no intra-axial or extra-axial masses. Under proper protocol, the patient was admitted to the operating room for biopsy. Intraoperatively, trachea and soft palate were displaced to the left. There was a large soft tissue mass located in the mandible, displacing her teeth all the way to the maxilla. The mass has expanded into the right neck area between sternocleidomastoid and trapezius muscles. Upon reflection of the mucoperiosteum on the lingual and buccal side, a soft tissue tumor was visible (Figure 1(e)). Careful dissection of the tumor from the lingual mucosa was performed removing a large specimen of approximately 6 × 6 × 3 cm, which was submitted for histopathology examination (Figure 1(e) (inset)). Further intraoperative examination revealed that the right mandible was completely destroyed and there was no more bone present. The tumor had expanded into the right neck region. All bleeding was stopped with monopolar and bipolar cautery. The patient underwent en bloc partial resection of right mandible. After removal of all soft tissue tumors, peripheral ostectomy was performed since part of the lesion involved the inferior alveolar nerve. The nerve was respected with immediate repair. Frozen section was done to rule out malignancy. After frozen section and resection, mandible was reconstructed with allograft. No tumor was left. Surgical incision was closed in a primary fashion. Once the patient stabilized, she was transferred to the Neonatal Intensive Care Unit (NICU). Histopathology examination revealed a tumor composed of spaces separated by septa (blood-filled cavities) (Figure 2(a)). The septa contained spindle cell proliferation with osteoclast-like giant cells and osteoid (Figures 2(b) and 2(c)). There were solid areas that show similar cellular components. Also, we detected numerous mitotic figures reaching more than 10/10 high-power field (HPF) (Figure 2(d)). The focal area at the periphery exhibits mineralized deposits accompanied by spindle cell proliferation. No histopathology evidence of Ewing sarcoma, ameloblastoma, or malignancy was seen. The histopathological diagnosis was mandibular aneurysmal bone cyst with cemento-ossifying fibroma. The patient had complete healing of her bony defect with significant resolution in her unique facial deformity that resembles sarcoma (Figure 1(f)). 46 months of her last follow-up, the patient is healthy, alive with no recurrence.

## 3. Discussion

ABCs are nonodontogenic, nonepithelial cysts that commonly occur in long bones and the spine. Van Arsdale was the first who described ABCs in 1893. Later in 1942, the term aneurysmal bone cyst was applied by Jaffe and Lichtenstein [7]. Only 2-3% of ABCs occur in the head and neck region, most of them (around 66%) occur in the jaw [5]. As compared to the maxilla, ABCs most frequently occur in the mandible. Our literature review found more than 92 reported cases of ABCs located in the jaw [5]. Most of these are multilocular, well-defined, and located in the mandible. The mean size was 4.8 cm. The recurrence rate of ABC in the jaw is considered lower than that of long bones. Similar recurrence rates were found in primary and secondary jaw ABCs. ABCs commonly recurred after 1 year of surgical resection and rarely after 2 years. Thus, it is recommended to follow up at least 2 years after resection [5]. The pathogenesis of ABC remains unclear. Theories range between primary or secondary lesion and a local abnormality within the bone. Suggestive causes of primary ABC are posttraumatic conditions (such as dental extraction), reactive vascular malformation, and genetically predisposed bone tumors. ABCs could be initiated due to a reactive body condition, such as increased venous pressure due to vascular malformation or circulatory disorder, which leads to high intraosseous venous pressure, bone expansion, and destruction of the vascular bed. Other studies have suggested that ABCs originate due to preexisting bone lesions, such as hemangioma, fibrous dysplasia, ossifying fibroma, central giant cell granuloma, or chondroblastoma [7]. Most of these theories share a common vascular pathogenesis. Recent studies proposed that primary ABCs are more toward a tumor origin than a reactive process. Genetic studies show a gain of chromosomal function in ubiquitin protease gene (USP6 orTre2) on chromosome 17p13(TRE17), leading to t(16; 17)(q22; p13). Osteoblast cadherin 11 (CDH11) on chromosome 16q22 is the most common fusion partner [8].

ABCs can also be categorized according to their clinical and radiological behavior as inactive, active, or aggressive. Clinical presenting symptoms are variable and nonspecific and might include pain, localized swelling, and pathologic fracture. Radiology workup has variable appearing morphology. Typically, it appears as a radiolucent multilocular cyst with ill-defined internal septations. Multidetector computed tomography (MDCT) and magnetic resonance imaging (MRI) might show the classic multiple cysts with fluid-fluid levels [8]. The most common type of ABC is the “classic or vascular form” (95%) that is composed of blood-filled cavities, sinusoidal spaces with hemosiderin-laden macrophages, and multinucleated giant cell separated by cellular fibrous connective tissue. The other less common form is the “solid” type (5%), which is a noncystic variant of ABC, composed mainly of solid soft tissue. Hemorrhage and fibroblastic spindle cell proliferation is often seen. Areas of osteoblastic differentiation with osteoid calcification can be seen. A third form also known as “mixed-type” contains solid and cystic areas [9]. The differential diagnosis includes telangiectatic osteosarcomas, cavernous hemangiomas, central giant cell granulomas (CGCGs), and brown tumors. ABCs can be radiologically confused with telangiectatic osteosarcoma, especially in cases of aggressive behavior and rapid growth. Telangiectatic osteosarcomas are a highly atypical tumor in which stromal cells are hyperchromatic with nuclear pleomorphism and atypical mitoses are present. Streamers of lace-like osteoid within the septa are also seen [10]. Unlike ABC, which reveals thick septa surrounded by blood-filled spaces that contain multinucleated giant cells and non-atypical stromal cells, no nuclear pleomorphism, hyperchromasia, or atypical mitosis is seen. Cavernous hemangiomas are large cystically dilated vessels with thin walls without cellular atypia [11]. CGCGs (also known as giant cell reparative granuloma) are lesions of the mandible and maxilla. Their soft tissue counterpart tumor is peripheral giant cell granuloma [12]. Brown tumors are multinucleated giant cells with abundant hemosiderin deposition due to hyperparathyroidism. The treatment of ABC is controversial, with a wide variety of acceptable therapeutic options. These include conservative treatment with frequent follow-up and simple curettage, cryotherapy, excision of the lesion, radical resection with reconstruction and bone grafts, or calcitonin and methylprednisolone lesion injection [7].

In conclusion, ABCs are benign osteolytic lesions that commonly appear in the first two decades of life, predominantly involving the mandibular body. Their clinical presentation can be atypical with large destructive mass, mimicking high-grade malignancy. It can cause huge swelling and compression symptoms. ABC should be considered in the differential diagnosis of maxillary bone lesions, and surgical curettage represents the main method of accurate diagnosis.
